# Distance teaching of experimental scientific methodology and scientific thinking

**DOI:** 10.3205/zma001411

**Published:** 2021-01-28

**Authors:** Alice Assinger, Matthäus Grasl, Ivo Volf

**Affiliations:** 1 Medizinische Universität Wien, Institut für Gefäßbiologie und Thromboseforschung, Vienna, Austria; 2 Medizinische Universität Wien, Universitätsklinik für Hals- Nasen- und Ohrenkrankheiten, Vienna, Austria; 3 Medizinische Universität Wien, Institut für Physiologie, Vienna, Austria

**Keywords:** distance teaching, scientific thinking and reasoning, flow cytometry, biochemical methods

## Abstract

**Objective: **The aim of this project was to convert a traditional face-to-face seminar for the teaching of experimental scientific methodology to remote teaching in a timely manner due to the COVID-19 related restrictions to teaching in presence.

**Methodology: **The main focus of the course was on flow cytometry. Basics were developed in a virtual presence phase. Specific teaching contents were taught by an interactive presentation, which came very close to the user experience of a flow cytometer and interactively illustrated the influence of different experimental conditions on the obtained results.

Video sequences of authentic sample acquisitions were integrated into Adobe Captivate^®^. These “virtual acquisitions” were not distinguishable from the original procedure. For interpretation of the resulting diagrams, interactions were inserted, which allowed direct comparison of the obtained results.

**Implementation:** A presentation with interactive elements and video sequences was created and used for the virtual presence phases. After publishing on a web server in HTML 5, contents were made available to the students for post-processing of learning contents by self-paced learning with full (interactive) functionality.

**Conclusion: **Contributions elaborated by the students during the course demonstrate a learning outcome comparable to that archieved in the last years in presence mode. While implementation of this solution represented a highly time-consuming process, narrative feedback was consistently positive. Due to the short time available for implementation, no systematic evaluation could be conducted, which represents a clear limitation of this work.

## Zusammenfassung

**Zielsetzung: **Die Zielsetzung des dargestellten Projekts bestand darin, ein Seminar zur Vermittlung experimentell- wissenschaftlicher Methodik zeitnah auf Distanzlehre umstellen. Anlass dafür war der COVID-19-bedingte Entfall der Präsenzlehre.

**Methodik: **Der inhaltliche Schwerpunkt der Lehrveranstaltung lag in der Durchflusszytometrie. Die Grundlagen dazu wurden in einer virtuellen Präsenzphase erarbeitet. Für die weiteren spezifischen Unterrichtsinhalte wurde eine interaktive Präsentation verwendet, welche dem Bedienerlebnis eines Durchflusszytometers sehr nahe kam und den Einfluss unterschiedlicher experimenteller Bedingungen auf die erhaltenen Ergebnisse interaktiv veranschaulichte.

Dazu wurden unter anderem Videosequenzen authentischer Probenaquisitionen in Adobe Captivate eingebunden. Diese „virtuellen Aquisitionen“ waren in ihrer optischen Darstellung vom Originalvorgang nicht unterscheidbar. Für die Interpretation der damit erhaltenen Diagramme wurden Interaktionen eingefügt, welche ein vergleichendes Gegenüberstellen der erhaltenen Ergebnisse ermöglichten.

**Umsetzung: **Eine Präsentation mit interaktiven Elementen und Videosequenzen wurde erstellt und in den virtuellen Präsenzphasen verwendet. Nach Veröffentlichung im HTML 5-Format stand sie den Studierenden für eine Nachbearbeitung der Lerninhalte in selbstbestimmter Ablaufsteuerung und voller (interaktiver) Funktionalität über das Internet zur Verfügung.

**Schlussfolgerung: **Die durch die Studierenden erarbeiteten Inhalte lassen ein inhaltliches Verständnis erkennen, welches mit dem der letzten Jahre (in Präsenzmodus) vergleichbar ist. Das erhaltene narrative Feedback war durchwegs positiv, der für die Implementierung dieser Lösung notwendige Arbeitsaufwand war unverhältnismäßig hoch. Aufgrund der Kurzfristigkeit der Umsetzung konnte keine systematische Evaluation durchgeführt werden, was eine klare Limitation dieser Arbeit darstellt.

## Introduction

The course presented here represents a compulsory elective course of the 10^th^ semester (seminar/practical course 809.086, Fluorescence-based methods in cell biological research) of 15 academic hours, which was held for 3 groups of 5 participants each within different weeks.

The learning objective was to promote scientific thinking. For this purpose, different biochemical methods were presented to the students. Based on selected problems, students had to discuss which scientific questions can (not) be answered by means of the respective methodology. In the practical part of the face-to-face course, students were instructed in the use of a flow cytometer (antibody staining of their own blood samples) followed by basal analysis and interpretation of the obtained results.

Due to the COVID-19 prohibition of classroom teaching, this course had to be adapted to remote teaching.

## Project description

**Figure 1:**
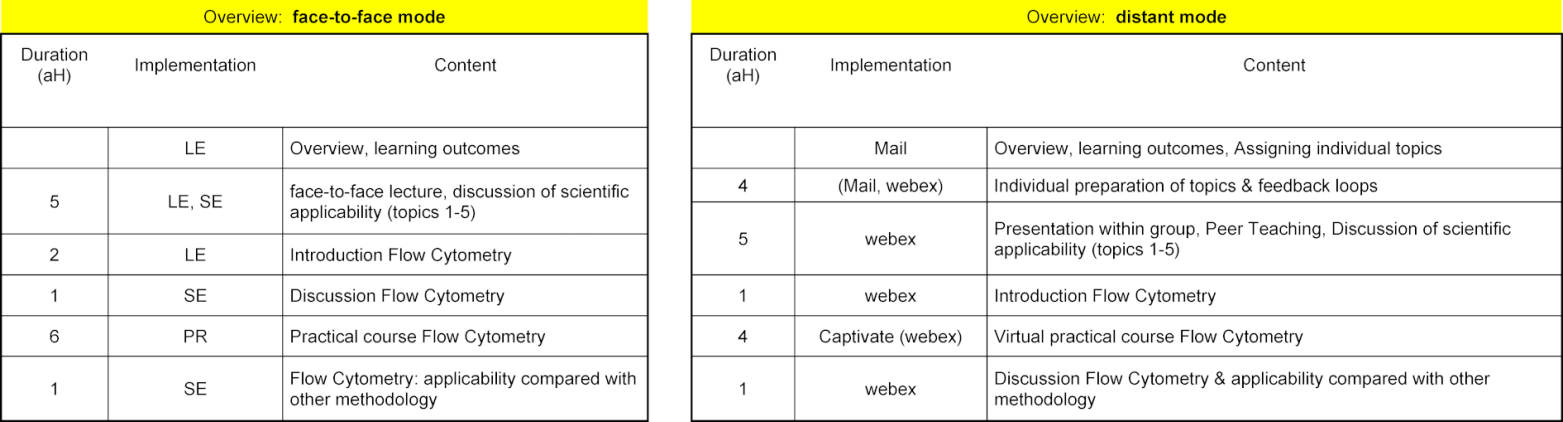
Schematic overview of the course in face-to-face mode (left) and distant mode (right). There were five topics to prepare in distant mode (antibodies, detection strategies, cell-cell interaction and inflammation, detection of proteins, microscopy: examination of cells). Students received detailed definition of topics and a selection of recommended sources (pdf, hyperlinks pointing to text- or video-based content). Students had to prepare a summary of the respective topic and considerations regarding the applicability on concrete scientific problems. Duration shown for the respective elements represents only the curricular relevant duration, the exact time that students required to prepare their topics can only be estimated. aH: academic hour (45 minutes); LE: Lesson; SE: Seminar; PR: Practical.

This was realized by assigning an individual topic to each of the five students of each group (see legend in figure 1 (Fig. 1)). While preparing these individual topics, students received feedback and advice regarding structure and intended level of detail of the presentation they had to prepare (maximum of 4 slides). In addition to a concise summary of the given topics, scientific thinking was promoted by the requirement to consider the applicability of the respective methodology to specific scientific questions (“Formulate different scientific questions which could be investigated with ...”).

For a subsequent virtual presence phase, a group jigsaw puzzle was used and the prepared topics were taught to the whole group in a (supervised) peer-teaching session. This was followed by a discussion on content, applicability of the methodology and the scientific questions proposed for the topics.

Since the main focus of the course was on applied flow cytometry, great efforts were made to compensate for the loss of face-to-face teaching – and the resulting inability to operate the cytometer by hand – as effective as possible.

Concrete goal was to make virtual sample acquisition appear as realistic as possible and to illustrate the influence of different experimental conditions on the results obtained.

The theoretical basics of flow cytometry were specifically taught in an independent virtual presence phase. A presentation created in Adobe Captivate^®^ was used for the subsequent cytometry-specific teaching content.

Video sequences of authentic sample acquisitions were integrated into Adobe Captivate^®^. After documentation and verbal explanations of the respective experimental conditions, this “virtual acquisition” could be started by pressing a button – the visual presentation of sample acquisition was not distinguishable from the original process. In addition, interactions were inserted for the interpretation of the (virtually) obtained scatter plots, which allowed to directly compare the plots obtained during sample acquisition (basal demonstration available on http://educativo.at/DemoFACS/)

This presentation was shared via WebEx from the screen of the lecturer with the students of the respective group; for the individual examination of the interactive elements as well as for post-processing of learning contents, the presentation was available to the students for self-paced learning and full (interactive) functionality on the internet.

## Methodology

A presentation with interactive elements and video sequences was created in Adobe Captivate^®^. This presentation was published on a web server in HTML 5 format and therefore was accessible from any web browser independent of the operating system (i.e., also on tablets).

For a user experience as close as possible to the original sample acquisition, video sequences of authentic sample acquisitions were recorded at a frame rate of 30 frames per second on the control computer of the flow cytometer. The software used was easyscreencastrecorder (freeware, [https://www.portablefreeware.com/index.php?id=2840]), which allows the recording of videos also in defined subareas of the screen. The resulting mpeg (Audio Codec Engine PCM) was converted with OpenShot (freeware, [https://www.portablefreeware.com/index.php?id=2840]) to mp4 (h.264) with video profile HDV 720 24p (1280x720) in high quality and integrated into Captivate in this format.

## Discussion

Adobe Captivate^®^ was used to create an interactive presentation that was also made available to students via the internet. Due to the short time available for the required implementation, no systematic evaluation could be conducted, which is a clear limitation of this work. Narrative feedback from students was consistently positive; contributions elaborated by the students during the course demonstrate a learning outcome comparable to that archieved in the last years in presence mode. 

Implementation of this solution represented a highly time-consuming process of about 35 hours for a resulting teaching time of 4 hours. The interactive course described here will also be implemented when returning to classroom teaching.

## Competing interests

The authors declare that they have no competing interests.

